# Should Pediatric Endocrinologists Consider More Carefully When to Perform a Stimulation Test?

**DOI:** 10.3389/fendo.2021.660692

**Published:** 2021-03-22

**Authors:** Arturo Penco, Benedetta Bossini, Manuela Giangreco, Viviana Vidonis, Giada Vittori, Nicoletta Grassi, Maria Chiara Pellegrin, Elena Faleschini, Egidio Barbi, Gianluca Tornese

**Affiliations:** ^1^ University of Trieste, Trieste, Italy; ^2^ Institute for Maternal and Child Health IRCCS “Burlo Garofolo”, Trieste, Italy

**Keywords:** endocrinologic diseases, stimulation tests, visit and budget of health, epidemiology, growth hormone deficiency, central precocious puberty, congenital adrenal hyperplasia, central adrenal insufficiency

## Abstract

**Introduction:**

Pediatric endocrinology rely greatly on hormone stimulation tests which demand time, money and effort. The knowledge of the pattern of pediatric endocrinology stimulation tests is therefore crucial to optimize resources and guide public health interventions. Aim of the study was to investigate the distribution of endocrine stimulation tests and the prevalence of pathological findings over a year and to explore whether single basal hormone concentrations could have saved unnecessary stimulation tests.

**Methods:**

Retrospective study with data collection for pediatric endocrine stimulation tests performed in 2019 in a tertiary center.

**Results:**

Overall, 278 tests were performed on 206 patients. The most performed test was arginine tolerance test (34%), followed by LHRH test (24%) and standard dose Synachthen test (19%), while the higher rate of pathological response was found in insulin tolerance test to detect growth hormone deficiency (81%), LHRH test to detect central precocious puberty (50%) and arginine tolerance test (41%). No cases of non-classical-congenital adrenal hyperplasia were diagnosed. While 29% of growth hormone deficient children who performed an insulin tolerance test had a pathological peak cortisol, none of them had central adrenal insufficiency confirmed at low dose Synacthen test. The use of basal hormone determinations could save up to 88% of standard dose Synachthen tests, 82% of arginine tolerance + GHRH test, 61% of LHRH test, 12% of tests for adrenal secretion.

**Conclusion:**

The use of single basal hormone concentrations could spare up to half of the tests, saving from 32,000 to 79,000 euros in 1 year. Apart from basal cortisol level <108 nmol/L to detect adrenal insufficiency and IGF-1 <-1.5 SDS to detect growth hormone deficiency, all the other cut-off for basal hormone determinations were found valid in order to spare unnecessary stimulation tests.

## Introduction

Children and young people referred to pediatric endocrinology services present with a wide range of illnesses and disorders varying from minor to life-threatening conditions (1). Diagnosis rely greatly on laboratory testing: while for some disorders a single blood sample is sufficient (e.g. primary hypothyroidism), in others (e.g. growth hormone deficiency [GHD] or central precocious puberty [CPP]) the determination of basal hormones is of limited diagnostic value, since many hormones are secreted in pulses or have specific oscillatory activity.

Theoretically, stimulation tests should more sensitively pick up disorders that would be missed by assessing spontaneous hormone concentrations as compared with basal hormone concentrations. Therefore, stimulation tests are used to assess the maximum secretion of a hormone and/or are as a proxy parameter of endogenous secretion, in order to evaluate if a child is producing enough or too much hormones compared to the normal functioning of endocrine system for age (2).

However, stimulation tests demand time, money and effort: they require the use of an intravenous line to inject the stimulating hormone and/or chemical substance and can take up to three or more hours; they need special staff skills, and are typically conducted in a hospital outpatient setting; for instance, in Italy the costs for stimulation test – which is free of charge for patients and families and covered by the Italian National Health System – vary from 305 to 591 euros, while a single hormone determination costs from 9.40 to 16.90 euros.

Many authors have proposed basal cut-offs in order to avoid unnecessary tests (3–7); this would help in sparing time, money and effort. Nevertheless, stimulation tests are still extensively used and considered the gold standard in the diagnosis of many endocrine diseases.

The knowledge of the pattern of pediatric endocrinology stimulation tests is therefore crucial to optimize resources and guide public health interventions; however, no studies have evaluated this topic comprehensively so far.

Aim of the study was to investigate the distribution of endocrine stimulation tests and the prevalence of pathological findings over a year and to explore whether single basal hormone concentrations could have saved unnecessary stimulation tests.

## Materials and Methods

We conducted a retrospective study at the Institute for Maternal and Child Health IRCCS “Burlo Garofolo” in Trieste, Italy, a tertiary hospital and research institute that serves as a pediatric referral center for the province of Trieste, and as national reference hospital.

All records of children and adolescents performing a stimulation test from January 1^st^ to December 31^st^ 2019 were reviewed. The “G2 clinico” platform (management system specialist activities) was employed to access all patients’ data. Information retrieved included age at presentation, gender, type and number of tests performed, reason(s) for referral, test results and final diagnosis.

Stimulation tests were performed according to protocols (8). In case of suspected non-classical-congenital adrenal hyperplasia (NC-CAH), a Standard Dose Synacthen Test (SDST) was performed and 17-hydroxy-progesterone (17-OH-P) data were interpreted according to New’s nomogram (9), while in case of suspected central adrenal insufficiency (CAI), a Low Dose Synacthen Test (LDST) was performed and a normal response was considered a peak cortisol level of ≥430 nmol/L (10–12); for peak values between 430 and 500 nmol/l, a rise in cortisol levels >200 nmol/l was used as confirmation of normal response (13).

In case of suspected central precocious puberty (CPP) or Hypogonadotropic Hypogonadism (HH), a Luteinizing Hormone Releasing Hormone Test (LHRHT) was performed and peak Luteinizing Hormone (LH) >5 IU/L were considered as pubertal (14).

In case of suspected growth hormone deficiency (GHD), Arginine Tolerance Test (ATT), Arginine Tolerance Test plus Growth Hormone Releasing Hormone Test (ATT+GHRHT) or Insulin Tolerance Test (ITT) were performed. ITT was considered valid only with biochemical hypoglycemia at a blood glucose level ≤40 mg/dl. According to Italian regulation, a peak plasma GH concentration of <8 ng/ml (<20 ng/ml if test is ATT + GHRHT) was considered diagnostic of GHD when confirmed in two tests performed in two different days (15). As second test, ITT was usually performed in order to exclude a concomitant adrenal insufficiency; when peak cortisol at ITT was <430 nmol/L and CAI was suspected (15), a LDST was performed for confirmation. To confirm GHD in the transition age (adult GHD, AGHD), an ATT+GHRHT was performed and a peak plasma GH concentration of <19 ng/ml was considered as pathological (16).

Cut-offs were taken into account to consider diagnosis without stimulation tests: for NC-CAH a basal 17-OH-P <1 (9) or <2 ng/ml (3); for CAI a basal cortisol <108 or >381 nmol/L (4); for CPP and HH a basal LH ≥1 IU/L (5); for GHD an IGF-1 ≥-1.5 SDS (6); for AGHD an IGF-1 ≥-1.7 SDS (7).

Ethical Committee approval was not requested since General Authorization to Process Personal Data for Scientific Research Purposes (Authorization no. 9/2014) declared that retrospective archive studies that use ID codes, preventing the data from being traced back directly to the data subject, do not need ethics approval (17). Informed consent was signed by parents at first visit, in which they agree that “clinical data may be used for clinical research purposes, epidemiology, study of pathologies and training, with the objective of improving knowledge, care and prevention”.

Statistical analyses were mainly descriptive. Data are presented as frequencies and percentages, or as median and interquartile ranges (IQRs) due to non-normal distribution. Receiver operating characteristic (ROC) analysis was used to evaluate the sensitivity and specificity of significant variables for predicting pathological tests. A P-value <0.05 was considered statistically significant. Analyses were performed with JMP™ software (version 15.1.0, SAS Institute Inc., Cary, NC, United States).

## Results

Overall, 278 tests were performed on 206 patients (119 females) with a median age of 11.1 years (IQR 8.0-14.0). While 146 individuals performed only 1 test (71%), 60 individuals (29%) performed 2 or more tests.

**Figure 1:**
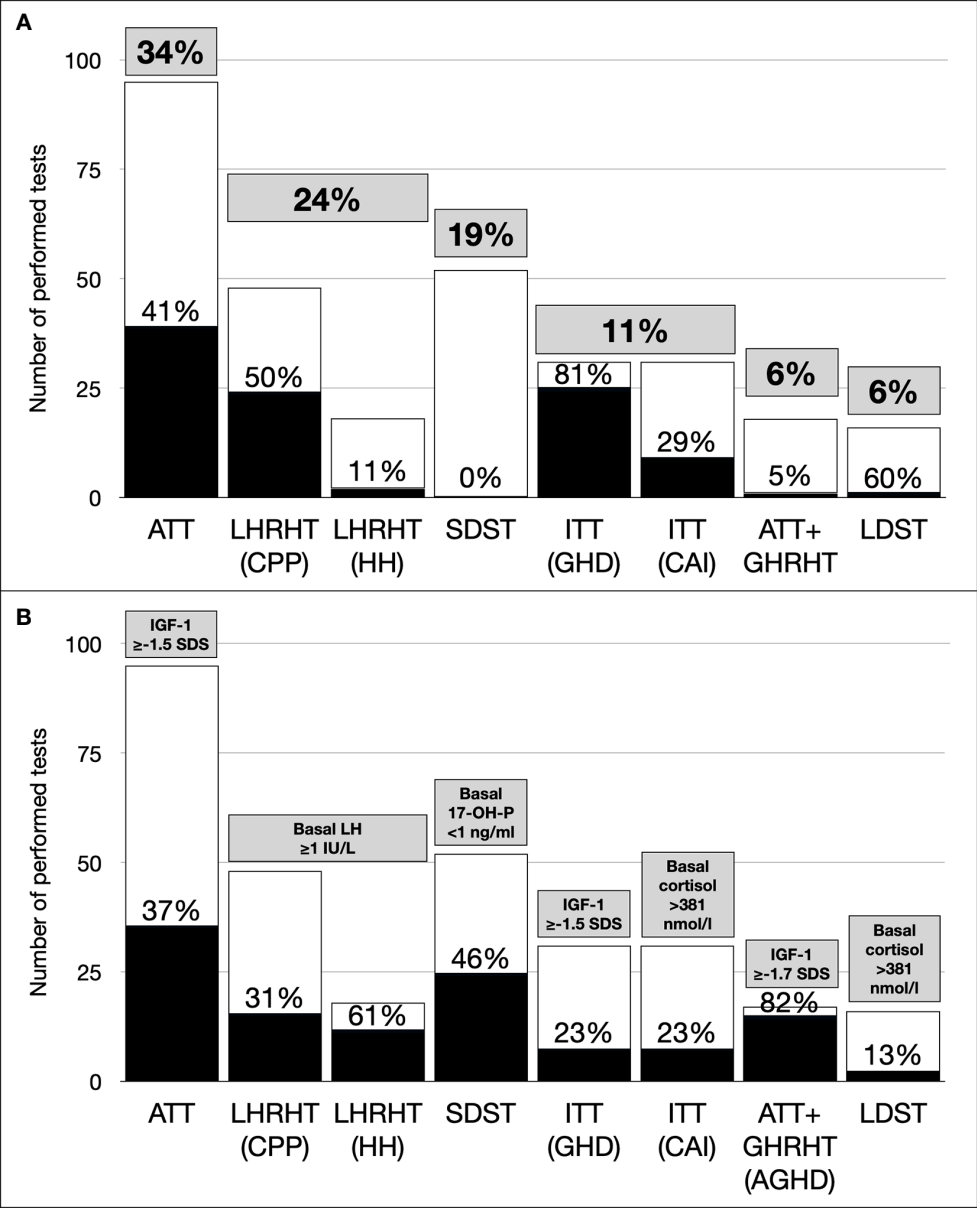
**(A)** Distribution of performed and pathological tests (grey boxes: prevalence on overall tests; dark bars: prevalence of pathological findings for each test). **(B)** Distribution of spared tests using single hormone determination (gray boxes, used cut-offs; dark bars, prevalence of saved tests for each test). 17-OH-P, 17-hydroxy-progesterone; AGHD, Adult Growth Hormone Deficiency; ATT, Arginine Tolerance Test; CAI, Central Adrenal Insufficiency; CPP, Central Precocious Puberty, GHD, Growth Hormone Deficiency; GHRHT, Growth Hormone Releasing Hormone Test, HH, Hypogonadotropic Hypogonadism; IGF-1, Insulin Growth Factor-1; ITT, Insulin Tolerance Test; LDST, Low Dose Synacthen Test; LH, Luteinizing Hormone; LHRHT, Luteinizing Hormone Releasing Hormone Test; NC-CAH, Non-Classical Congenital Adrenal Hyperplasia; SDS, Standard Deviation Score; SDST, Standard Dose Synachten Test.

The distribution of stimulation tests and of pathological response are reported in **Table 1** and graphically in **Figure 1**.

**Table 1:** Distribution of stimulation tests and suspected diagnosis with number and rate of performed and pathological tests, number and rate of spared tests according to different cut-offs, and number and rate of false negatives using cut-offs (*all individuals did not confirm pathological results at LDST).

Stimulation test	Suspected diagnosis	N of performed tests	% on all tests	N of pathological tests	% of pathological tests	Cut-off	N of spared tests	% of spared tests	N of false negative	% of false negative
*ATT*	GHD	95	34%	39	41%	IGF-1 ≥ −1.5 SDS	35	37%	9	26%
*LHRHT*	CPP	48	24%	24	50%	Basal LH ≥ 1 IU/L	15	31%	0	0%
	HH	18		2	11%	Basal LH ≥ 1 IU/L	11	61%	0	0%
*SDST*	NC-CAH	52	19%	0	0%	Basal 17-OH-P <1 ng/ml	24	46%	0	0%
						Basal 17-OH-P <2 ng/ml	46	88%	0	0%
*ITT*	GHD	31	11%	25	81%	IGF-1 ≥ −1.5 SDS	7	23%	5	71%
	CAI			9	29%*	Basal cortisol > 381 nmol/l	7	23%	0	0%
						Basal cortisol < 108 nmol/l	2	6%	1	50%
*LDST*	CAI	16	6%	1	6%	Basal cortisol > 381 nmol/l	2	13%	0	0%
						Basal cortisol < 108 nmol/l	2	13%	2	100%
*ATT + GHRHT*	AGHD	17	6%	0	0%	IGF-1 ≥ −1.7 SDS	14	82%	0	0%
	GHD	1		1	100%	IGF-1 ≥ −1.5 SDS	1	100%	1	100%

17-OH-P, 17-hydroxy-progesterone; AGHD, Adult Growth Hormone Deficiency; ATT, Arginine Tolerance Test; CAI, Central Adrenal Insufficiency; CPP, Central Precocious Puberty; GHD, Growth Hormone Deficiency; GHRHT, Growth Hormone Releasing Hormone Test; HH, Hypogonadotropic Hypogonadism; IGF-1, Insulin Growth Factor-1; ITT, Insulin Tolerance Test; LDST, Low Dose Synacthen Test; LH, Luteinizing Hormone; LHRHT, Luteinizing Hormone Releasing Hormone Test; NC-CAH, Non-Classical-Congenital Adrenal Hyperplasia; SDS, Standard Deviation Score; SDST, Standard Dose Synachten Test.

### Adrenal Function Tests

In 52 cases (40 females) SDST was performed to rule out NC-CAH (median age 9.1 years [IQR 7.8-14.3]). No cases of NC-CAH were confirmed, while in 45 individuals, results were compatible with carrier status.

Twenty-four individuals with a basal 17-OH-P <1 ng/ml would have been correctly classified as not having NC-CAH without performing the stimulation test (**Figure 1B**) or 46 individuals with a cut-off of <2 ng/ml.

Forty-nine tests (LDST or ITT) to explore adrenal sufficiency were performed in 38 individuals (17 females), with a median age of 12.1 years [IQR 10.0-13.6].

In 31 individuals (13 females), ITT was performed as second test to investigate GH secretion.

Thirteen individuals had a peak cortisol at ITT <430 nmol/L (4 of which performed the ITT in 2018) and performed a LDST as confirmation of CAI: the peak cortisol was >430 nmol/l in all the subjects, while it was <500 nmol/l in 6 subjects with a rise in cortisol levels >200 nmol/l in all (median 272 nmol/l [IQR 246-301]).

In 3 individuals (2 females), LDST was performed because CAI was the main diagnostic hypothesis and in 1 CAI was actually diagnosed.

In 9 cases (7 ITT, 2 LDST), basal cortisol was already >381 nmol/l (**Figure 1B**). No individuals had basal cortisol <100 nmol/l, while in 4 cases (2 ITT, 2 LDST) basal cortisol was <108 nmol/l and all of them resulted not have CAI at stimulation tests.

### Puberty Tests

In 48 individuals (38 females) LHRHT was performed to investigate CPP (median age 7.8 years [IQR 7.2-8.9]) and CPP was confirmed in 24 children (16 females). All 15 children (9 females) with a basal LH ≥1 IU/L would have been correctly diagnosed with PPC without performing the stimulation test (**Figure 1B**).

In 18 individuals (11 females) LHRHT was performed to investigate HH (median age 14.5 years [IQR 13.1-15.8]). HH was confirmed in 2 individuals (1 female). The 11 individuals (7 female) with a basal LH ≥1 IU/L would have been correctly classified as not having HH without performing the stimulation test (**Figure 1B**).

In our cohort, a pubertal response (peak LH >5 IU/L) was associated with higher basal LH (median 1.2 vs. 0.3 mUI/ml; p<0.01). At ROC analysis, a basal LH value >0.2 identified the highest percentage of correctly classified (true positives and true negatives), with sensitivity and specificity related to the identification of pubertal response of 97% and 60%, respectively.

### Growth Hormone Stimulation Tests

**Figure 2:**
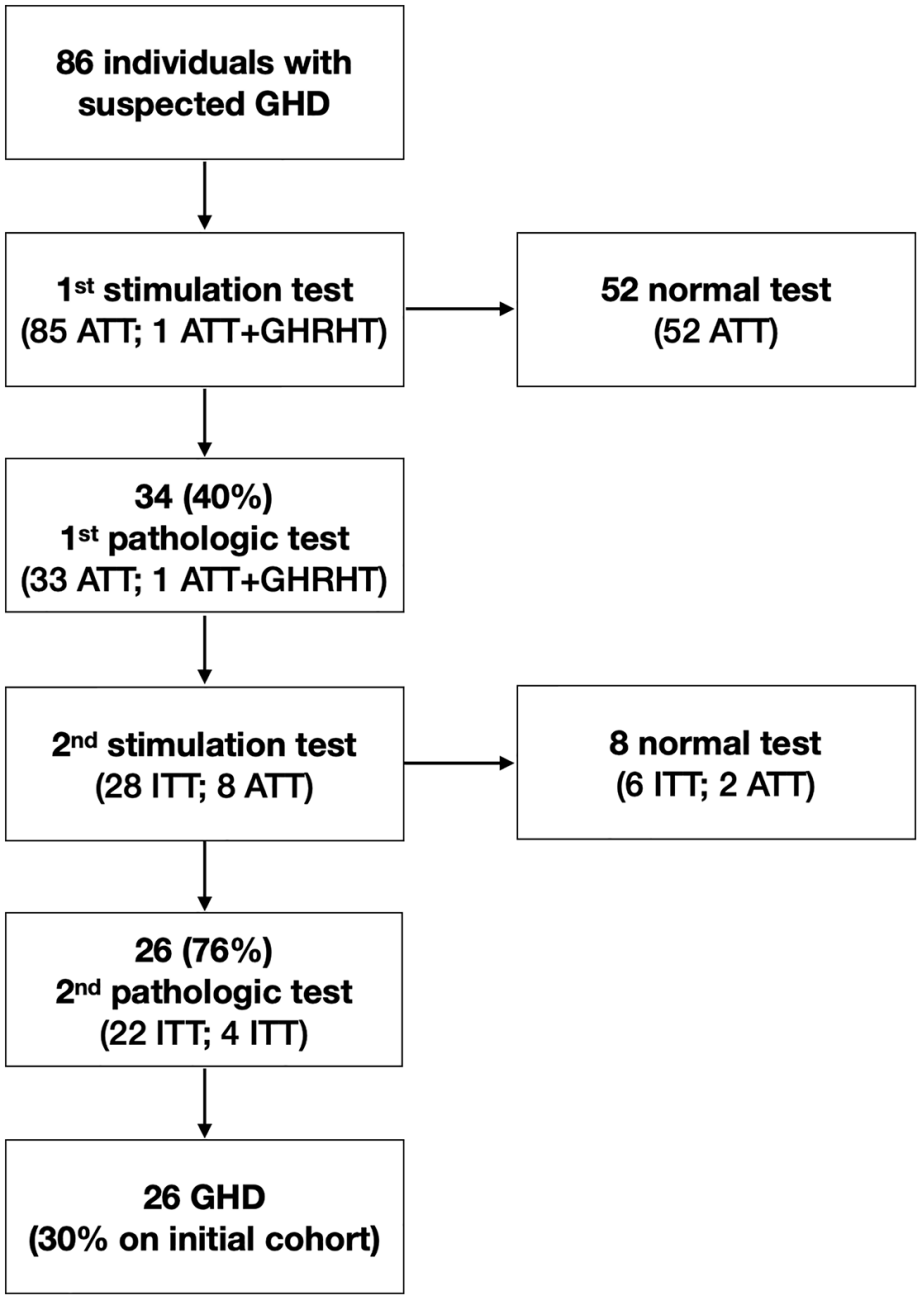
Diagram of test performed to confirm suspected growth hormone deficiency (ATT, Arginine Tolerance Test; GHD, Growth Hormone Deficiency; GHRHT, Growth Hormone Releasing Hormone Test; ITT, Insulin Tolerance Test).

Among 86 individuals who completed their evaluation for suspected GHD during the study period, the first stimulation test was pathological in 34 individuals (76%); of these, 26 (76%, 30% when considering the initial cohort) had a second pathologic stimulation test, having GHD confirmed (see **Figure 2** for details on performed tests).

A pathological response (GH peak <8 ng/mL) was associated with lower IGF-1 SDS (median -2.1 vs. -1.6; p=0.01). At ROC analysis, an IGF-1 SDS <-1.5 identified the highest percentage of correctly classified (true positives and true negatives), with sensitivity and specificity related to the identification of GHD patients of 79% and 52%, respectively.

Using a cut-off of IGF-1 ≥-1.5 SDS for not performing a stimulation test, 43 test would have been spared (**Figure 1B**); however, 6 individuals would have been not diagnosed with GHD.

None of 17 patients (6 females, median age 17.2 years [IQR 16.6-17.7]) with GHD in childhood and tested in transition age resulted to have an AGHD.

The 14 individuals (4 female) with IGF-1≥-1.7 SDS, would have been correctly classified as not having AGHD without performing the stimulation test.

## Discussion

In this retrospective study, we comprehensively analyzed 278 tests performed on 206 patients in a tertiary pediatric endocrinology over 1-year period. To our knowledge, this is the first study that comprehensively considered all endocrine stimulation tests at the same time and showing the potential impact of basal hormone cut-offs on sparing tests.

Our study confirmed that basal hormone determinations can be a useful first-line step to screen patients requiring stimulation tests. We demonstrated that the use of cut-offs suggested by literature could save a great amount of stimulation tests: up to 88% of SDST, 82% of ATT+GHRHT, 61% of LHRHT, 12% of tests for adrenal secretion. Overall, using single basal hormone concentrations, we could have been spared from 113 to 135 tests (41-49%), saving from 32,000 to 79,000 euros.

Some of these cut-offs, however, are still not sure enough to exclude or confirm a diagnosis without performing a stimulation test.

To make a definitive diagnosis of CAI, several basal morning cortisol cut points have been suggested [<80 (18), <100 (19–22), <108 (4), <138 (23), <230 nmol/l (24)]. In order not to lose diagnosis, we used the lowest possible cut-off (108 nmol/l, since no individuals had basal cortisol <100 nmol/l); nonetheless, in our cohort a basal cortisol under this threshold was not associated with CAI at stimulation tests. As a matter of fact, although a low basal cortisol strongly suggests adrenal dysfunction, still no guideline support the use of basal cortisol to diagnose CAI (25).

Analogously, the single use of IGF-1 SDS could have caused a missed diagnosis in 6 over 26 patients with GHD (23%). This confirm that IGF-1 measurement has poor accuracy in discriminating children with or without GHD, since also those with IGF-1 in the normal range (>-1.5 SDS) could be diagnosed with GHD. Therefore, IGF-1 values should not be used alone in the diagnosis of GHD, but always be interpreted in combination with other clinical and biochemical parameters (6).

In our cohort, the most performed test was ATT (34%), followed by LHRHT (24%) and SDST (19%), while the higher rate of pathological response was found in ITT to detect GHD (81%), LHRHT to detect CPP (50%) and ATT (41%), apart from ATT+GHRHT that was performed in only 1 obese individual to detect GHD and resulted pathological. Interestingly, all SDST performed to exclude NC-CAH and all ATT+GHRHT performed to exclude AGHD resulted negative.

Regarding the tests to investigate growth hormone secretion, the rate of confirmed GHD on all tests was 30% and only 76% of the first positive growth hormone stimulation tests had a second positive result. The rate of false positive results in the first test is in line with an extensive study conducted on 472 children with normal stature who underwent provocative tests to assess GH secretory status (26). The study reported false positive rates between 8.9% and 23.7%, according to the test performed, with a GH cutoff <7 ng/dl. In an Italian single-center retrospective study conducted on 166 children with a first pathologic ATT test, GHD was confirmed in 80.2% of patients (27), thus resulting in accordance with our study. Therefore, in the Unites States and almost all the Europe, two stimulation tests are required in order to reduce the risk of false positive results (28). The ITT test is still considered the gold standard with a high power of discrimination. The combination of ITT with a second pharmacological stimulation test allows the highest power of discrimination in detecting GHD (29).

Interestingly, while 29% of GHD children who performed an ITT had a peak cortisol <430 nmol/l, none of them had CAI confirmed at LDST. In our center, a peak cortisol of 430 nmol/l was used as threshold for both tests as per protocol (8) and according to literature and used methodology (10–12, 15), although different cut points have been suggested for both tests up to 500-600 nmol/l (30, 31). When borderline peak cortisol levels (430-500 nmol/l) were found, a rise in cortisol levels >200 nmol/l was used to confirm normal adrenal function (13).

The evaluation of adrenal axis in patients with GHD is of primary importance to exclude combined pituitary hormone disorders; even if asymptomatic, the identification of patients with subclinical CAI is of utmost importance to avoid life-threatening events secondary to stressful circumstances (32). Moreover, the introduction of growth hormone replacement may unmask both an incipient adrenal insufficiency, besides central hypothyroidism (33). An inappropriate cortisol response to ITT is considered the gold standard in detecting CAI (34); however, LDST is a reliable test in patients with CAI since the adrenal gland cannot response to ACTH stimulation when there is insufficient endogenous ACTH. In our cohort, patients with pathological ITT and normal LDST peak cortisol response did not started hydrocortisone replacement treatment and no child developed CAI on growth hormone treatment; however, the progression towards CAI in unclear and follow-up is needed.

NC-CAH was excluded in all the patients tested (the prevalence of NC-CAH is 1:800-1:1,000), whereas 87% were identified as possible carriers of 21-hydroxylase deficiency. This means that in a selected cohort, referred for precocious adrenarche, the prevalence of carriers might be extremely higher than in general population (9.5%) (35). However, knowing the status of carriers is not of value in pediatric age, since no treatment is needed and genetic testing is not allowed (36).

A potential limitation of this study is based on data collected from a single-center, therefore results may be related to the local population. On the other hand, to our knowledge, this is the first study that has simultaneously analyzed the features of pediatric endocrine stimulation tests as a whole and the potential save of money by using basal hormone concentrations as screening before performing a test.

## Conclusion

The study provides information on the distribution of endocrine stimulation tests and the prevalence of pathological findings over a year. Apart from basal cortisol level <108 nmol/L to detect adrenal insufficiency and IGF-1 <-1.5 SDS to detect GHD, all the other cut-off for basal hormone determinations were found valid in order to spare stimulation tests. Nonetheless, in order not to miss a diagnosis, it is always important to consider the overall clinical picture that led to biochemical investigation and to follow-up patients where a doubt exists.

## Data Availability Statement

The raw data supporting the conclusions of this article will be made available by the authors, without undue reservation.

## Ethics Statement

Ethical review and approval was not required for the study on human participants in accordance with the local legislation and institutional requirements. Written informed consent to participate in this study was provided by the participants’ legal guardian/next of kin.

## Author Contributions

AP and BB conceptualized and designed the study, drafted the initial manuscript, designed the data collection instruments, collected data, and reviewed and revised the manuscript. MG carried out the statistical analysis, and reviewed and revised the manuscript. VV, GV, and NG performed the stimulation tests, and reviewed and revised the manuscript. MP, EF, and EB conceptualized and designed the study and critically reviewed the manuscript for important intellectual content. GT conceptualized and designed the study, carried out the statistical analysis, drafted the initial manuscript, coordinated and supervised the data collection, and reviewed and revised the manuscript. All authors contributed to the article and approved the submitted version.

## Conflict of Interest

The authors declare that the research was conducted in the absence of any commercial or financial relationships that could be construed as a potential conflict of interest.
